# Atypical MRI features in familial adult onset Alexander disease: case report

**DOI:** 10.1186/s12883-016-0734-9

**Published:** 2016-11-04

**Authors:** Yonghong Liu, Heng Zhou, Huabing Wang, Xiaoqing Gong, Anna Zhou, Lin Zhao, Xindi Li, Xinghu Zhang

**Affiliations:** Department of Neurology, Beijing Tiantan Hospital, Capital Medical University, No 6 Tiantanxili, Dongcheng District, Beijing, 100050 China

**Keywords:** Alexander disease, Adult onset, Familiar, GFAP, Gene mutation

## Abstract

**Background:**

Alexander disease (AxD) is a rare neurological disease, especially in adults. It shows variable clinical and radiological features.

**Case presentation:**

We diagnosed a female with AxD presenting with paroxysmal numbness of the limbs at the onset age of 28-year-old, progressing gradually to spastic paraparesis at age 30. One year later, she had ataxia, bulbar paralysis, bowel and bladder urgency. Her mother had a similar neurological symptoms and died within 2 years after onset (at the age of 47), and her maternal aunt also had similar but mild symptoms at the onset age of 54-year-old. Her brain magnetic resonance imaging (MRI) showed abnormal signals in periventricular white matter with severe atrophy in the medulla oblongata and thoracic spinal cord, and mild atrophy in cervical spinal cord, which is unusual in the adult form of AxD. She and her daughter’s glial fibrillary acidic protein (GFAP) gene analysis revealed the same heterozygous missense mutation, c.1246C > T, p.R416W, despite of no neurological symptoms in her daughter.

**Conclusions:**

Our case report enriches the understanding of the familial adult AxD. Genetic analysis is necessary when patients have the above mentioned symptoms and signs, MRI findings, especially with family history.

## Background

Alexander disease (AxD, OMIM 203450) is a rare but fatal central nervous system disease. Three subtypes are distinguished upon the onset age: infantile (under age 2), juvenile (age 2 to 12) and adult (over age 12). The infantile form is the most common subtype while the adult onset the least. All subtypes have been described and present with different clinical manifestations. However, the pathological hallmark of the disease is the accumulation of ubiquitinated intracytoplasmic inclusions in astrocytes, called rosenthal fibers, which are composed of glial fibrillary acidic protein (GFAP), the main intermediate filament of astrocytes [1].

The adult cases can be distinguished in familial or sporadic form. Here, we present a case of adult onset AxD with family pedigree.

## Case presentation

A 34-year-old female was evaluated at our hospital with a 6-year history of progressive neurological symptoms. At age 28, she developed paroxysmal numbness in the left limbs, and two years later she had unilateral limb weakness. She was misdiagnosed as multiple sclerosis at the other hospital 2 years after onset. After high-dose pulse methylprednisolone therapy, the illness had no mitigation. The above mentioned symptoms progressively worsened. At age 31, she subsequently developed ataxia, dysarthria, depression, and bowel and bladder urgency with occasional incontinence. And her hands became mild muscle atrophy one year later.

**Fig. 1:**
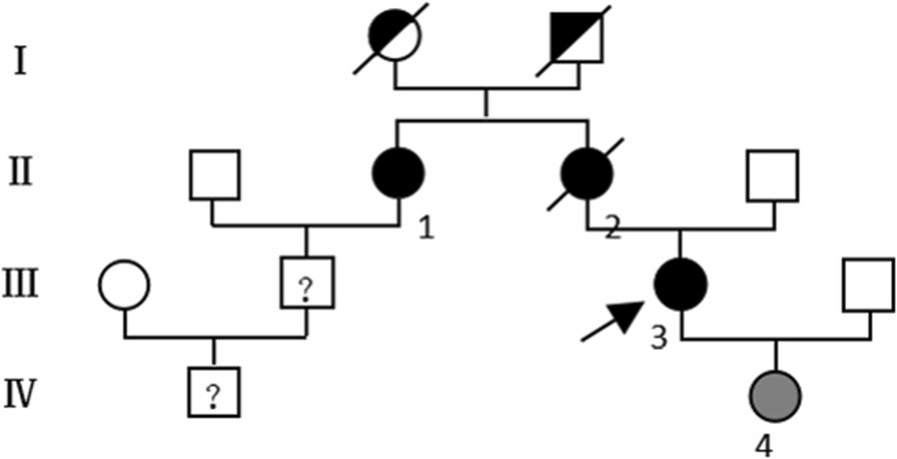
Family pedigree. *Black filled symbols* represent patients. *Grey filled symbols* refer to patient’s daughter who had GFAP mutations but without neurological signs or symptoms. *Empty symbols* represent healthy subjects. *Half-filled symbols* refer to presumably affected ancestor. *Question marks* mean subjects potentially affected without neurological signs or symptoms, not examined nor tested for GFAP mutations. *Oblique slash* means the deceased. *Black arrow mark* represents the propositus (our patient). Patient III-3 and her daughter (patient IV-4) performed the molecular diagnosis. Although the family member (II-1) had similar psychological manifestations, the detailed medical record was not obtained and not examined for GFAP mutations

She denied any medical history before. Her mother had a similar neurological symptoms and died within 2 years after onset (at the age of 47), and her maternal aunt also had similar but mild symptoms at the onset age of 54 years old. Her 8-year-old daughter didn’t present with any neurological symptoms but had brain lesions. Other family members were healthy (Fig. 1).

Her general medical examination was unremarkable. In neurological examination, she had normal mental status and language testing. The mixed spastic-ataxic dysarthria and horizontal gaze nystagmus were noted. Myokymia was observed in lingualis with weakness of bulbar muscles. Her hands showed mild muscular atrophy. The muscles strength of the bilateral upper-extremities, the left lower-extremity, the right lower-extremity were grade 4, 2, 1, respectively. The increased tendon reflexes and the positive Babinski signs were observed bilaterally. Ataxia was observed in bilateral upper limbs. There was no apparent sensory deficit or extrapyramidal signs. The latest score of modified Rankin scale is 4.

Serum laboratory studies showed unremarkable (complete blood count, routine chemistry test, clotting studies, electrolyte panel, creatinine, glucose, liver and renal function tests, vitamin B12, vitamin E, folic acid, autoimmune antibodies, and infection including hepatitis virus antibodies, Treponema serology, HIV antibodies). Cerebrospinal fluid examination showed a normal opening pressure, the cell count, protein, glucose, IgG index, anti-aquaporin-4 antibody, and intrathecal IgG synthesis rate without oligoclonal bands.

Electroencephalogram, electrophysiological, visual evoked potential, as well as all of the autonomic nervous system testing were all normal 2 years after onset except aural conduction in their brainstem evoked potential.

**Fig. 2:**
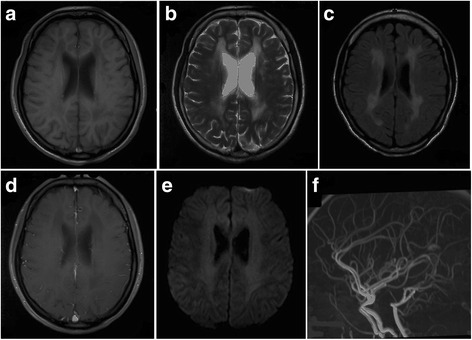
Axial Brain MRI showed low T1-weighted signals (**a**), high T2-weighted signals (**b**), and hyperintensity in FLAIR image(**c**) around the periventricular area. MRI with contrast enhancement was normal (**d**). DWI revealed slight hyperintensity (**e**). MRA were normal (**f**)

**Fig. 3:**
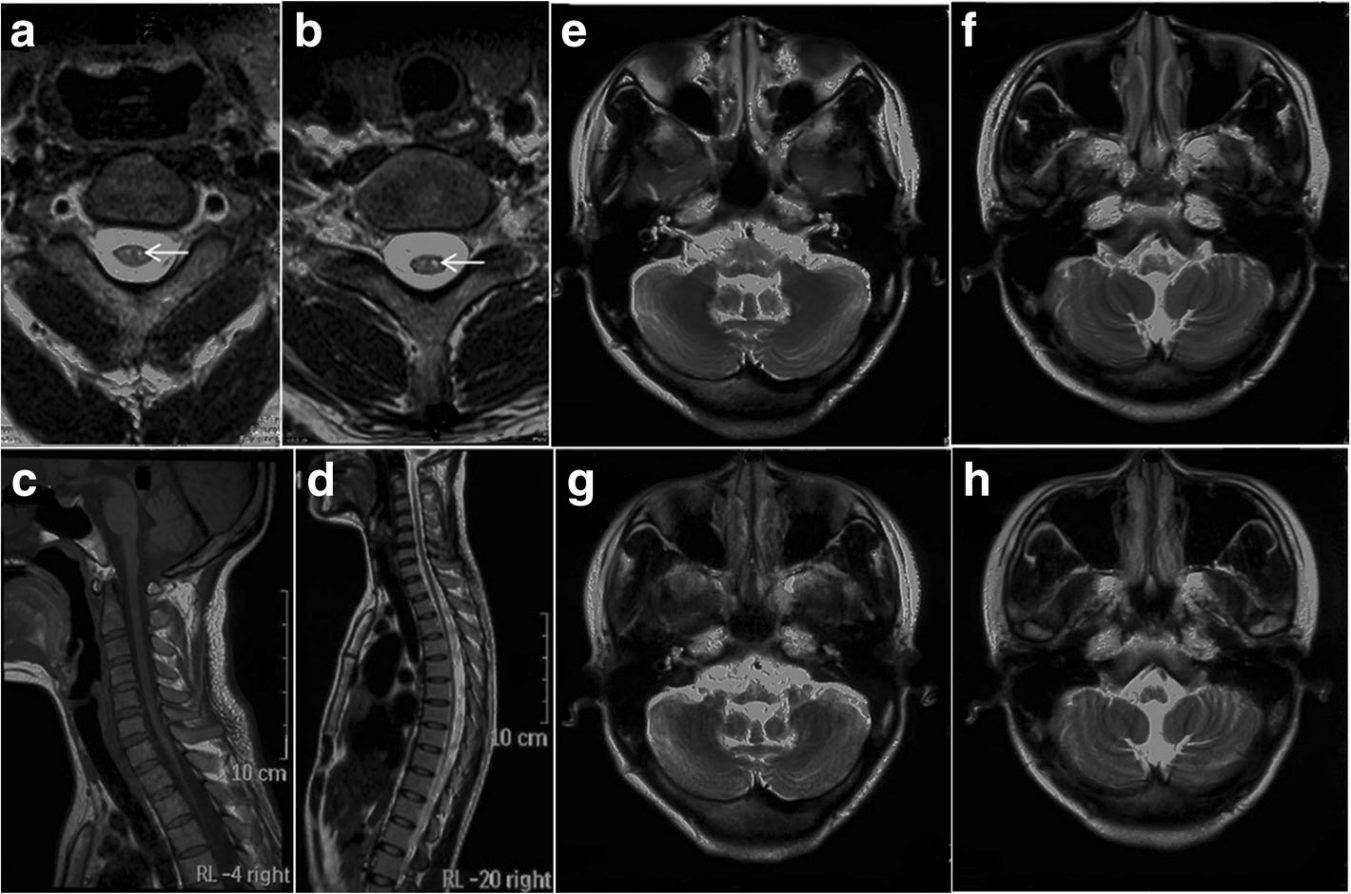
Axial spinal MRI showed hyperintensity involving the corticospinal tracts extends from the carotid 2 (**a** *arrowhead*) to carotid 7 cord (**b** *arrowhead*) in T2WI image, mild atrophy of cervical spinal cord in sagittal T1WI image (**c**), severe atrophy of whole thoracic segments in sagittal T2WI image, which is similar to the typical “tadpole” (**d**), medullary atrophy at 3 years after onset (**e**, **f**), and severe medulla and cerebellar atrophy 1 years later (**g**, **h**)

**Fig. 4:**
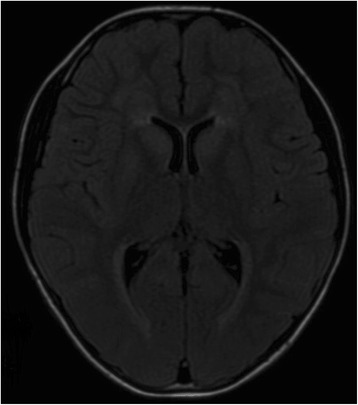
Axial FLAIR image of the asymptomatic daughter of our patient show band-like hyperintense lesions around the lateral ventricle

Her brain MRI demonstrated bilateral and symmetric abnormal signals without gadolinium enhancement, which were predominantly distributed in the periventricular, and subcortical white matter. Diffusion Weighted Imaging (DWI) revealed slightly hyperintensity in the periventricular areas. Magnetic resonance angiography was normal [Fig. 2]. The marked atrophy of the medullary oblongata and thoracic spinal cord was seen, while the atrophy of cervical spinal cord was relatively mild. Abnormal T2 hyperintensity was noted in the pyramidal tract of spine. With symptoms progressively worsening, medullary and cerebellar atrophy accelerated [Fig. 3]. Her daughter brain MRI showed abnormality in the periventricular white matter [Fig. 4].

**Fig. 5:**
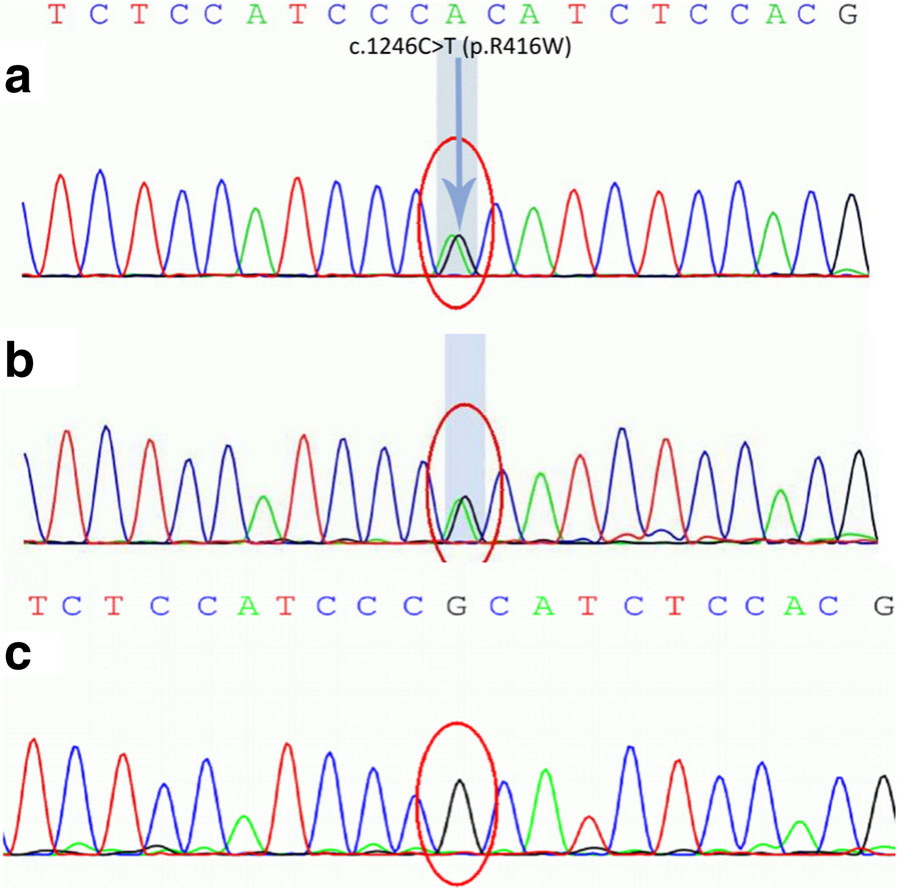
The chromatograms of sequencing results. **a** The patient. **b** The daughter. **c** The control. In the area of the GFAP gene exon 8, there is a heterogeneous missense mutation point: c.1246C > T (cytosine > thymine, *Aarrowhead*), leading to amino acid change in p.R416W (arginine > tryptophan)

Her genomic DNA was extracted from the peripheral leucocytes when an informed consent was obtained from the patient and her husband. A heterogeneous missense mutation was detected in exon 8 (c.1246C > T) of the GFAP gene, causing a change of arginine to tryptophan at amino acid position 416 (p.R416W). Her daughter’s genomic DNA showed the same heterozygous mutation but without any neurological symptoms [Fig. 5]. Upon clinical, family history, MRI finding and gene analysis, she was confirmed as adult AxD.

This case with AxD is characterized by typical clinical and MRI findings, especially with family history and adult onset. The heterozygous missense mutation in the GFAP gene, c.1246C > T, p.R416W was identified, which was already shown to be pathogenic in adult onset AxD [2, 3]. It has been reported that adult AxD has various clinical courses. Most cases have a subacute onset and gradually progressive course [2], but some present an acute onset [4]. There is no gender difference. About half of the cases is familial, consistent with autosomal dominant transmission. The mean age of onset is usually in the late thirties. Our patient had pseudobulbar, spastic paresis and ataxia, which are acknowledged as the cardinal triad of the clinical presentations and appear in approximately 70 % of cases [2, 4, 5]. Palatal myoclonus, which is specific and essential for AxD diagnosis, is only observed in one third, especially in hereditary cases [6], not seen in our patient. Nearly 45 % of reported patients had autonomic dysfunction including bowel/bladder dysfunction and orthostatic hypotension [7], which was found in our patient.

Typical MRI findings included marked signal periventricular changes, atrophy of the bulbar region and upper cervical spinal cord in adult AxD [2, 7]. Approximately 90 % cases showed marked medullary atrophy and 50 % had deep white matter lesions or periventricular changes [5]. Farina et al. reported that patients under 40 years old were easier to have periventricular white matter abnormalities and postcontrast enhancement than patients over 40, which showed different levels of abnormal diffusivity On DWI, but some cases were normal [8]. In our case, MRI findings showed the severe atrophy in bulbar region and thoracic spinal cord, but mild atrophy in cervical spinal cord, which is rare in the adult AxD. On DWI, our patient had slightly hyperintensity of extensive periventricular white matter. The lesions without gadolinium enhancement might be related to the decreased amounts and limited distribution of rosenthal fibers [8]. It should be stressed that MRI findings in our case was atypical despite of the lesion locating in the typical regions.

In the radiologic differential diagnosis of adult AxD, several disorders need to be considered [9]. Diseases like degenerative disorders, particularly progressive supranuclear palsy, multisystem atrophy with cerebellar predominance, and various spinocerebellar ataxias often have brain stem and cerebellum atrophy but without lesions in periventricular white matter [8]. Besiedes, some leukodystrophies which are similar to adult AxD were described below (see Table 1) [5, 10–17].

**Table 1:** The characteristics of common leukodystrophies in adult

Disease	Clinical presentation	Abnormal regions or abnormalities on brain MRI	Gene mutations
X-ALD	SP	CST, dorsal columns, CC, PWM	ABCD1
Metachromatic leukodystrophy	PP, motor impairment	bilateral frontal PWM, CC; cortical atrophy	ARSA
Krabbe’s disease (GALC)	SP or tetraparesis, PDPN, CD, seizures, cortical blindness	Supratentorial, CWM, PT, splenium of CC and optic radiation, CST; CC atrophy	GALC
VWM (CACH)	migraine, PP, dementia, PBP, SP	enlargement of the lateral ventricles; WM	EIF2B
HDLS/POLD	behavioral changes, dementia, motor impairment, epilepsy	internal capsules, CST; WM with non-enhancing; frontal lobes atrophy	CSF1R
ADLD	AS, BBD, OH, PS, ataxia	frontoparietal WM, CP, CST, CC	LMNB1
Cerebrotendinous xanthomatosis	PP, SP, CA, polyneuropathy, tendon xanthomatas	dentate nucleus, CWM, CP, PT, PWN, CC, basal ganglia; brain and cerebellar atrophy	CYP27A1
NHD/PLOSL	PP, memory loss, dementia, skeletal abnormalities	nonspecific WM; cortical atrophy	TREM2 DAP12
CADASIL	migraines, TIA, strokes, PP, CD	PWM in the centrum semiovale, external capsules and anterior temporal poles	NOTCH3
CARASIL	TIA, strokes	diffuse WM changes, lacunar infarcts	HTRA1

*X-ALD* X-linked adrenoleukodystrophy, *VWM* Vanishing white matter disease, *CACH* childhood ataxia with central hypomylination, *HDLS* hereditary diffuse leukoencephalopathy with neuroaxonal spheroids, *POLD* Autosomal dominant pigmentary type of orthochromatic leucodystrophy, *ADLD* Adult-onset autosomal dominant leukodystrophy, *NHD* Nasu-Hakola disease, *PLOSL* polycystic lipomembranous osteodysplasia with sclerosing leukoencephalopathy, *SP* spastic paraparesis, *PP* psychiatric problems, *PBP* pseudobulbar palsy, *WM* white matter, *CWM* cerebellar WM, *PT* pyramidal tracts, *CD* cognitive decline, *AS* autonomic symptoms, *BBD* bowel and bladder dysfunction, *OH* orthostatic hypotension, *PS* pyramidal symptoms, *CA* cerebellar ataxia, *TIA* transient ischemic attacks, *PDPN* peripheral demyelinating polyneuropathy, *CST* corticospinal tracts, *CC* corpus callosum, *PWM* periventricular WM, *CP* cerebellar peduncles

Beside, asymptomatic carriers with MRI abnormalities have been described in some reports [2, 6, 8, 18–23]. Here we listed several asymptomatic carriers similar to our patient’s daughter who had abnormal brain MRI and carrying the gene mutation (see Table 2).

**Table 2:** The information of asymptomatic carriers with MRI abnormalities

Patient Reference	Gender	Age (years)	MRI scan	Nucleotide change	Aminoacid substitution
Patient 1 [18]	Male	62	Mild cervicomedullary atrophy	c.232G > A	p.D78N
Patient 2 [6]	Male	32	Atrophy of medulla oblongata and spinal cord, Periventricular rim	c. 274 T > G	p.V87G
Patient 3 [19]	Male	33	Atrophy of medulla oblongata and spinal cord, white matter lesion, periventricular rim	c. 274 T > G	p.V87G
Patient 4 [20]	Male	^a^<4	Frontal white matter abnormality	c.276C > T	p.R88C
Patient 5 [2]	Male	30	Supratentorial periventricular white matter, atrophy of medulla oblongata and cervical cord	c.613G > A	p.E205K
Patient 6 [21]	Male	32	Periventricular rim, atrophy of medulla	c.988C > G	p.R330G
				c.994G > A	p.E332K
Patient 7 [22]	Female	34	Mild abnormal intensities in the deep frontal white matter and caudates	c.1006 T > C	p.L331P
Patient 8 [23]	Male	72	Atrophy of the upper cervical cord medullaoblongata and cerebellar	c.1157A > G	p.N386S
Patient 9 [8]	Male	52	Supratentorial periventricular white matter, atrophy of medulla oblongata and cervical cord	c. 1193C > A	p.S398Y
Patient 10 [20]	Male	^a^<11	White matter of the cerebellum, medulla, pons changes	c.1260C > T	p.R416W
				c.154C > T	p.P47L

^a^No age at onset is reported for Patients 4 and 10; evaluation for leukodystrophy was initiated only after incidental findings of white matter changes were discovered by MRI performed as part of examination for other conditions

Mutations in the GFAP gene, which lead to the accumulation of ubiquitinated intracytoplasmic inclusions in rosenthal fibers in association with the small heat shock proteins, HSP27 and aB-crystallin [1], are thought to account for more than 95 % of AxD cases [24]. In infantile [3, 25, 26], juvenile [20, 27] and sporadic adult AxD [28–30], the heterozygous missense mutation of the GFAP gene, c.1246C > T, p.R416W, has been reported to be one of the causes of AxD. While In familial adult onset AxD it has been rarely reported. Thyagarajan described a woman and her son with adult onset AxD and the above mutation [31]. They had different clinical manifestations but strikingly similar MRI abnormalities and the same GFAP mutation. It means that molecularly characterized inherited AxD is a cause of symptoms. The same GFAP mutation can cause both early and late onset AxD, and vertical transmission occurs in adult onset AxD with GFAP mutations. Our patient, her mother and maternal aunt had similar neurological manifestations but the latter two had a relative later onset. Nevertheless, her mother’s clinical manifestations deteriorated rapidly, her maternal aunt has mild symptoms with comparatively slow progress. She and her daughter had the same heterozygous missense mutation. All these findings suggest that the familial Adult AxD members can possibly show either similar or different manifestations. The molecular genetic evidence of our AxD family favors that AxD is an autosomal dominant inheritance [32], but further long-term follow-up is needed.

## Conclusions

In summary, our case report enriches the understanding of the familial adult AxD. Genetic analysis is necessary when patients have the above mentioned symptoms and signs, MRI findings, especially with family history.

## Abbreviations

AxD: Alexander disease
DNA: Deoxyribonucleic acid
DWI: Diffusion Weighted Imaging
FLAIR: Fluid attenuated inversion recovery
GFAP: Glial fibrillary acidic protein
HIV: Human immunodeficiency virus
MRA: Magnetic resonance angiography
MRI: Magnetic resonance imaging
T1WI: T1-weighted imaging
T2WI: T2-weighted imaging
